# Web-Based Specialist Support for Spinal Cord Injury Person's Care: Lessons Learned

**DOI:** 10.1155/2012/861860

**Published:** 2012-08-15

**Authors:** Vincenzo Della Mea, Dario Marin, Claudio Rosin, Agostino Zampa

**Affiliations:** ^1^Medical Informatics, Telemedicine and eHealth Laboratory, Department of Mathematics and Computer Science, University of Udine, Via delle Scienze 206, 33100 Udine, Italy; ^2^Spinal Unit, Department of Rehabilitation Medicine, Physical Medicine and Rehabilitation Institute, 33100 Udine, Italy

## Abstract

Persons with disability from spinal cord injury (SCI) are subject to high risk of pathological events and need a regular followup even after discharge from the rehabilitation hospital. To help in followup, we developed a web portal for providing online specialist as well as GP support to SCI persons. After a feasibility study with 13 subjects, the portal has been introduced in the regional healthcare network in order to make it compliant with current legal regulations on data protection, including smartcard authentication. Although a number of training courses have been made to introduce SCI persons to portal use (up to 50 users), the number of accesses remained very low. Reasons for that have been investigated by means of a questionnaire submitted to the initial feasibility study subjects and included the still easier use of telephone versus our web-based smartcard-authenticated portal, in particular, because online communications are still perceived as an unusual way of interacting with the doctor. To summarize, the overall project has been appreciated by the users, but when it is time to ask for help to, the specialist, it is still much easier to make a phone call.

## 1. Introduction

Persons with disability from spinal cord injury (SCI) are subject to high risk of pathological events and need a regular followup even after discharge from the rehabilitation hospital [1]. SCI causes sensory, motor, and autonomic impairments, but often in long-term also a variety of secondary conditions on different domains, for example, physical (bladder and bowel problems, pain, spasms, pressure sores, and sexuality), psychological (anxiety and depression), and social (transport, finance, equipment, housing, care management, and employment).

SCI is not a static condition, but rather a process of continuous adaptation due to interactions with the aging process. Advanced age has been associated with increases in a number of secondary health complications including bowel complications, cardiovascular and respiratory complications, pressure ulcers, urinary tract infections, renal stones, and musculoskeletal pain.

Like in other countries, also in the Italian health-care system the GP is the first contact point for the person in need of health care. The same is true for people with SCI, encouraged to contact first their GP for health problems, but the limited expertise on SCI of the GP was seen by persons with an SCI as the greatest barrier to needs being met [1]. Thus most of SCI persons turn to either specialists of spinal units or rehabilitation centers, whose intervention is often made less effective by the following:

- distance between specialistic centers and SCI persons' home, so that they access them only for the most severe complications;
- scarce communication between specialistic centers and both GPs and generic hospitals to which SCI persons turn to because closer to home;
- small number of beds available for re-hospitalisation in specialistic centers.

Further critical issues include

- often inadequate planning of shared care paths between hospital and community-based health services;
- imprecise definition of roles and duties among spinal unit professionals and territorial healthcare service professionals.

As noted by Vaidyanathan et al. [2], also in the Italian health care system there are “artificial barriers in communication due to hierarchical or bureaucratic set up.” Authors also foresee that “good communication between spinal cord clinicians, patients, carers, and community health professionals by telephone, e-mail, or conventional postal system is likely to improve the care of spinal cord injury patients after discharge from spinal injury centres.”

In the last years an increase in the usage of telemedicine facilities has been recorded not only to enhance access to specialistic treatment by patients located far from specialized healthcare centers, but also to guarantee a higher efficiency of the healthcare system [3].

SCI patients have been in particular the subject of a number of telemedicine programmes aimed at ensuring continuity of rehabilitation after discharge and to prevent the most frequent and dangerous complications by means of in-home telephone or video-based interventions. Telemedicine in the followup of SCI persons include telerehabilitation [4, 5] as well as specific clinical evaluation practices like ulcer assessment. While there is evidence concerning the efficacy and effectiveness of telerehabilitation, evidence on economic aspects is still needed.

In addition to that, a fair number of disabled people is using information and communication technologies and, in particular Internet, to retrieve healthcare information and to directly communicate with the rehabilitation hospital care team [6]. Patient portals [7] and personalized health records [8, 9] are among the web applications considered of importance for the management of chronic conditions [10], as reported for example in diabetes [11].

In the two specialists authors experience, and in agreement with what reported in the literature [12], typical questions asked to the hospital team after discharge, either by phone or email, include small healthcare problems like

- prevention or treatment of urinary tract infections,
- counselling of bowel and bladder management,
- prevention of pressure ulcers,
- coordinating comprehensive care in a multidisciplinary team,
- continuity of care between primary and specialistic center, and
- support and education of the patient and his family.

Although the telephone and email are easy and often effective in facilitating communication between patient and specialist, it should be paid attention to at least three relevant problems:

- the GP is almost excluded from the communication and thus also from the care of his/her own patient;
- simply using standard electronic mail does not fulfill legal requirements according to the most recent regulations in matter of privacy and security, and most of the people are not yet using privacy-enhanced mail [13];
- due to the features of the communication medium, it is difficult to maintain a trace of all communications exchanged between patient and specialist.

Aim of the present work was to investigate whether a web portal fulfilling legal requirements can be used to mediate communications between SCI persons and their reference doctors (GP and rehabilitation specialist). For this, a web portal has been developed with the support of user groups and tested in a first feasibility study. After that, it has been deployed into the healthcare network of the Region Friuli-Venezia Giulia and provided to a larger number of users.

## 2. Methods

### 2.1. The System

The design of the system has been carried out in a shared initiative among rehabilitation specialists of the Spinal Unit of the regional rehabilitation hospital, representatives of users coming from the Regional Association of Para- and Tetraplegic people, and representatives of general practitioners. A work group has thus been established that supported the design, development, and testing of the proposed portal.

Among the specifications collected in the preliminary phase, the most important were those cited in the introduction, that is, compliance with law, involvement of GPs, and persistence of communications.

The system has been designed to be compliant with current W3C (World Wide Web Consortium) recommendations and regulations on accessibility [14] and with the requirement of future deployment into the Regional Healthcare Network.

The result has been a web-based system, where the core of communications involve three categories of actors (plus the latter, foreseen but not yet implemented):

- the person with a SCI condition;
- the GP of the person;
- the rehabilitation specialist of the spinal cord unit;
- the figure of caregiver, either a relative or friend, has been foreseen for the cases where direct access of the SCI person to the computer is difficult.

**Figure 1:**
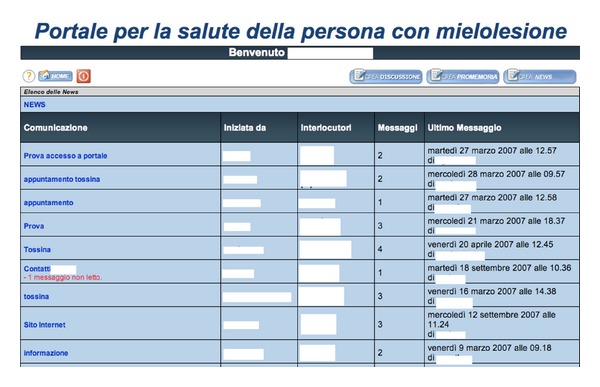
The web portal main communication interface.

The model of communication is simple. Communications occur in a forum-like interface based on threads (Figure 1), but with private communications among the three actors. To respect the initial requirements, any communication directed by the SCI person to the rehabilitation specialist is also automatically addressed to his/her own GP. This way, GP remains informed about his/her patient conditions. Doctors may also communicate directly without involving patients. In order to stimulate participation, all users may propose a theme of common interest for all users community, which is made public after moderation of a doctor. A small agenda module was also implemented for setting up appointments of patients.

The system has been then developed using ASP (Active Server Pages) (Microsoft, USA) technology and MySQL (Oracle, USA) and initially deployed on a research server in the Medical Informatics, Telemedicine and eHealth Laboratory at the University of Udine. The choice was directed towards future compatibility with the Regional Healthcare Network.

In the initial feasibility study, access control was implemented by means of username and password; however, the system was ready for more law-compliant approaches, by exploiting the existing infrastructure of the Regional Healthcare Network and its Citizen Portal.

### 2.2. Preliminary Experimentation

The experimentation, approved in an internal board review, included two phases:

- a first feasibility study with a small subject group;
- a second implementation phase with enrollment open among interested persons.

For the feasibility study, a subject group has been selected among those known as having computer and network facilities at home. Selection was carried out in a joint cooperation between the specialists involved in the study and the Association for Para- and Tetraplegic people. Subjects were also interviewed by the specialists (AZ and CR) to verify degree of disability, and at home by the scientist involved in the study (VDM) to verify computer knowledge level, to check which kind of facilities they had, and eventually to request the help of occupational therapists for solving accessibility or ergonomics problems. The GPs of selected subjects were then contacted to present the project and to invite them in the experimentation. In the initial phase, two specialists (coauthors of the present article) were directly involved too.

The preliminary experimentation has been carried out for six months, during which the portal has been iteratively enhanced according to user suggestions. Data about usage has been collected during this period, including number of communications and involvement of various parties.

### 2.3. Integration into the Regional Healthcare Network

After the initial study, which drove to minor modifications in the interface, the system has been transferred to the infrastructure of the Regional Healthcare Network and in particular into the Citizen Portal.

In the Friuli-Venezia Giulia Region there is in fact a network connecting all healthcare delivery points, from hospitals to small mountain wards, and there is also an infrastructure of software for internal sharing of data. In addition to that, recently a common Citizen Web Portal for region-related activities has been developed, that allows secure access to regional citizens according to current Italian laws in matter of privacy and security. This includes the use of a personal smartcard for client-side authentication, which has been freely distributed to all population, with a free reader upon request. The smartcard is issued at regional level but under national regulations, and serves at first as an administrative identification means when accessing healthcare services like hospitalization, medical visits, exams, and so forth. If “activated,” that is, associated to the person data after in-person identification by a public official, it becomes legally adequate for authentication.

However, healthcare content in the Citizen Portal is not yet fully developed, and at present only vaccinations lists, medical visits and exams bookings, and GP changes are available; so the Portal is not yet commonly used, although technically ready. In any case, activation of the smartcard for portal use is made upon reading and signing of an informed consent for giving the legal possibility of accessing citizen data by healthcare professionals.

Deployment on the regional network has been easy because the rest of the portal is developed in ASP, leaving out only the reimplementation of the user authentication basing on smartcard. Integration has been made by the regional software enterprise in charge of the healthcare network.

Users were then given an smartcard reader and instructed on its use.

Consequently, a informational and training programme has been set up to train interested subjects and invite them to use the Portal.

Not having seen a substantial usage of the Portal, after two years since the beginning of the project a multiple choice questionnaire has been submitted to the initial group of users. Questions asked are reported in Table 1.

Due to the low number of subjects, answers were not quantitatively analysed but only considered for discussion of the overall experiment.

## 3. Results

### 3.1. The Subjects

A group of 13 (4 paraplegics, 9 tetraplegics; 12 male, 1 female) persons with SCI has been selected initially for feasibility study; age ranged from 30 to 50 years at the time of enrollment, with an average history of SCI of 7 years. 8 of them have complete injury (class A according to ASIA classification), 5 incomplete (of which 2 ASIA B, 3 ASIA C). Only one was living alone, the others in their families.

All subjects were able to efficiently interact with their own home computer, either directly or by means of assistive products. The latter were mostly commercial, but sometimes also artigianally made or personalised from existing products, and included voice recognition software, substitutes to mouse to make it movable with the whole arm, sip and puff mouse buttons, eye tracking interface.

Each user signed an informed consent that specified also the modality of use of the portal, including the mandatory use of other channels for urgent needs, the 24 working hour response time, and the unavailability on weekends.

After the initial study, participation has been opened, and the system has been advertised during in-person training sessions carried out in the Rehabilitation Hospital or the Rehabilitation residential facility managed by the Regional Association of Para- and Tetraplegic People. Training involved features of the Citizen Portal as well as of the SCI portal too. Trainees willing to use the portal were then invited to use the Portal and to bring an explanation letter to their own GP.

In addition to that, the Association did their own advertisement through their journal, and organized two public meetings to describe the project.

50 SCI persons participated to a total of 8 training sessions. After each training session, subjects were proposed to be enabled for access to the portal. 23 of them had a home computer,were interested, and accepted. The other 27 were not interested in using the system or did not own a home computer.

### 3.2. Usage

Of 36 persons in total enabled to access the portal, 20 participated in at least one communication session on the web portal; 6 from the initial pilot group, 14 from the second phase. The number of doctors involved in discussions is substantially lower. In fact, while two rehabilitation specialists were involved in the project since the beginning and participated in communications, of the 20 GPs only 3 actually accessed the system.

The total number of communication threads has been 43, all initiated by SCI persons, for a total of 148 messages, of which 28 threads totalling 65 messages were coming from the pilot user group. 54 messages were answers from the hospital specialists; 5 from GPs. Thread themes were always small healthcare problems not needing an urgent access to healthcare facilities, including the following:

- bladder and bowel management;
- neuropatic pain treatment options;
- urinary infections, including laboratory exams interpretation;
- orthostatic hypotension;
- drugs assumption modalities and side effects;
- mattresses, wheelchair and cushion prescriptions;
- pressure ulcers conservative management;
- administrative requests (certificates, etc.).

However, since SCI is a complex condition, often threads regarded more than one of the above-mentioned topics, because of their interaction.

Although participating into the experimentation, 5 SCI persons contacted their rehabilitation specialists only by phone and/or email one or more times, even for nonurgent problems that could have been managed through the Portal.

No urgent questions were dealt through the Web Portal.

### 3.3. The Questionnaire

A 15 items questionnaire has been submitted to the SCI persons initially selected for the feasibility study, to understand the reasons why they used or not the portal, and what were its perceived advantages or problems. 12 out of 13 persons answered to questions (92%). The first four questions were aimed at understanding computer capabilities and attitudes; the others were specific to the project. A translation of the questionnaire is presented in Table 1, together with number of answers.

## 4. Discussion

Access to local healthcare facilities, including GP, is one of the major pitfalls to which is faced the person with SCI after discharge from rehabilitation hospital [1]. Thus such persons keep on considering the rehabilitation team and in particular the rehabilitation specialist as a reference point for most of their healthcare needs even after discharge [15].

This behaviour is facilitated also by the difficulties encountered by unspecialized healthcare personnel and GPs to be adequately trained in how to assist this peculiar class of healthcare consumers, taking into consideration the low prevalence of the condition [16].

The presented project has been aimed at overcoming the above mentioned issues, and thus

- to make easier for the SCI person to contact primary care givers like his/her own GP;
- to introduce a novel three-way communication habit among SCI person, specialist, and GP;
- start a sort of field training for GPs by means of the forum, where they could look at actions taken by the specialist when dealing with SCI persons.

The results of the experimentation however denote that we were not successful in our attempt.

Although portal usage was always meant only as a support for small healthcare problems, a critical analysis shows some crucial issues:

- the Web portal, while collecting positive and sometimes enthusiastic opinions when presented to SCI persons, has been not considered a primary choice when in need for communication with the rehabilitation specialist, thus preferring phone or even email;
- GPs did not show real involvement in the Portal;
- On the other side, the Portal has been correctly used for dealing with healthcare questions that could be treated in the asynchronous, nonurgent modality which is the only possible way through a web portal.

Due to their scarce involvement, no attempt has been made to submit a questionnaire to general practitioners.

The questionnaire submitted to the initial subject group revealed an overall appreciation of the system, although also helped in recognising some possible limitation of it. In fact, while not severely judged, usability of the web portal and of the smartcard authentication system seem to be the weak points of the project. Usability and user-friendliness have been recognised one of the possible issues for novice users of health portals [17, 18]. On the other side, security of communications has been reported not to be a concern of patients accessing portals [19], with the suggestion of providing more education on privacy and security.

However, smartcard is the technical method chosen at a national level for providing authenticated access in future web-based applications, in most part of the country not yet available. A couple of issues can be considered. First of all, the general public is not yet trained in understanding the need for privacy when accessing Internet and information systems, so the extra effort of using a smartcard with a PIN to be remembered and a card reader, which in turn needs to be installed, is not considered as something really needed.

Furthermore, the amount of effort depends also on the advantages provided by it. As the Citizen Portal is currently not providing many services to users, probably an application like the presented SCI Portal alone (which by definition should be used only when really needed) is not sufficient to trigger interest. In fact, at the time of writing, in the Region Friuli Venezia Giulia only a minority of people activated the card for web access, due to scarce interest in the provided services; an increase is foreseen when exam reports will be made available on the Portal. From this point of view, the selected group of people participating in the present study acted as early experimenters of a technology not yet widely used in the general population.

The availability of exam reports will also surely increase the interest in the limited mobility population including SCI persons, because a clear advantage will be provided in terms of reduced travels to take paper reports; at that time it is likely also the SCI portal will attract more participation.

Among the solutions suggested by subjects, the most important is the simplest one: more time is needed to become used to a novel communication modality between patient and doctor. This is because the main cause of scarce use is recognised to be the advantage of telephone over web portal, in terms of familiarity. Furthermore, Zickmund et al. reported that disinterest in a health portal is linked to satisfaction with the provider-patient relationship, including provider communication/responsiveness, and fear of losing relationships with the provider. [19]. Since the specialist-patient relationship in the considered scenario is good, maybe this also helped in limiting a concrete interest in the portal. 

To summarize, the overall project seems a good idea; it will be useful for supporting people with the same kind of problems, but when it is time to ask help to the specialist, it is still much easier to make a phone call, even if it has to be repeated many times in order to find the doctor.

Basing on the latter considerations, we decided to maintain the support system alive, waiting for other Citizen Portal applications (and in particular availability of laboratory reports) able to make to portal more popular among users. Reports and test results have been reported as the most used sections of an Internet Portal [20, 21]. In fact, a package of useful services might help in both pushing people (including SCI persons) in using the portal, as well as to maintain knowledge on how to use it due to a more frequent usage than what might be for the SCI persons support system alone. Intermittent usage does not help in developing loyalty to the system, and what Crutzen et al. defined “e-loyalty” [22] is a recognised issue for Internet interventions [23].

The main limitation of the present study is due to the users being selected because already owning and using a computer. The selected sample might be not fully representative of the whole population from the technological expertise point of view as well as from the health and mobility capabilities. Nijland et al. [18] suggested also that barriers of long term use may include selective enrollment: in our case, patients selected has having a computer could be also younger, educated and more autonomous in their procurement of health information, although we do not have data to support this hypothesis.

Even considering these limitations, the presented Portal might be seen as a first step towards the development of services able to mediate communication between citizens and their healthcare providers, with an active participation of the “e-patient” [24] into the healthcare management process, according to those principles that begin to be called “Medicine 2.0” [25].

## Figures and Tables

**Table 1:** The questionnaire and subject answers.

Questions and answer set	Number of answers
(a) When do you use the computer?	
(1) Every day	8
(2) some time a week	4
(3) once a week	
(4) some time a month	
(5) never	
(b) why do you use the computer? (max 3 answers)	
(1) to work	8
(2) to play	
(3) for Internet access	11
(4) for entertainment	4
(5) to communicate with others	6
(c) Do you think nowadays is important to know how to use the computer?	
(1) Yes, it is indispensable	8
(2) Yes, but depends on the job you do	4
(3) No, it is not necessary	
(4) No, it is a waste of time	
(5) I do not know	
(d) How do you judge your computer expertise?	
(1) Very good	
(2) Good	4
(3) almost good	4
(4) sufficient	2
(5) insufficient	
(6) bad	
(e) How do you judge the overall quality of the project?	
(1) Completely insufficient	
(2) insufficient	1
(3) sufficient	3
(4) good	4
(5) excellent	4
(f) How do you judge the usefulness of the project?	
(1) Completely insufficient	
(2) insufficient	
(3) sufficient	2
(4) good	6
(5) excellent	4
(g) How usable is the web portal?	
(1) Completely insufficient	
(2) insufficient	1
(3) sufficient	5
(4) good	6
(5) excellent	
(h) How do you judge the specialist answers quality?	
(1) Completely insufficient	
(2) insufficient	1
(3) sufficient	1
(4) good	6
(5) excellent	4
(i) How do you judge the time waited for answers?	
(1) Completely insufficient	
(2) insufficient	1
(3) sufficient	5
(4) good	2
(5) excellent	4
(j) How do judge the usability of smart card authentication?	
(1) Completely insufficient	
(2) insufficient	
(3) sufficient	4
(4) good	6
(5) excellent	2
(k) How did you feel about privacy using smart card?	
(1) Completely insufficient	
(2) insufficient	
(3) sufficient	6
(4) good	6
(5) excellent	
(l) In your opinion, why the web portal has been used so little?	
(1) No time	
(2) unfamiliar with technique (computer versus phone)	12
(3) unsatisfactory answers	
(4) answers difficult to understand	
(5) other	
(m) Which of the following solutions might help to enhance the usage of the tool?	
(1) computer training	
(2) a specific course to learn the use of web portal	4
(3) agreed upon time for answer	2
(4) more time to get used in a novel communication modality between patient and doctor	10
(5) making the web portal easier to use	2
(6) Other	
(n) Do you think that after an experimentation phase, this tool might become useful for people with the same problems as you?	
(1) Yes	12
(2) No	
(o) Do you think a connection between rehabilitation specialists and you general practitioner, about your health status, is useful?	
(1) Yes	12
(2) No	
